# Hypertrophic Cardiomyopathy Diagnosed on Point-of-Care Echocardiogram in an Elderly Patient With Syncope

**DOI:** 10.7759/cureus.17008

**Published:** 2021-08-08

**Authors:** Christopher M Stoll, Matthew Carr, Leily Naraghi

**Affiliations:** 1 Emergency Medicine, Maimonides Medical Center, Brooklyn, USA; 2 Emergency Medicine, Orange Park Medical Center, Brooklyn, USA

**Keywords:** hypertrophic obstructive cardiomyopathy, hocm, ultrasound, pocus echocardiogram, syncope, elderly

## Abstract

A bedside echocardiogram in the emergency department can provide a wealth of information about a patient’s hemodynamic status, anatomical structures, and response to medical interventions. This readily available tool can drastically guide management within minutes as soon as the undifferentiated critically ill patient enters the hospital. In this clinical scenario, we report a case of hypertrophic obstructive cardiomyopathy (HOCM) that was diagnosed for the first time in an elderly male, who was brought to the emergency department after a syncopal episode, utilizing bedside ultrasound and it highlights the significance of considering a broad differential.

## Introduction

Hypertrophic obstructive cardiomyopathy (HOCM) often goes undiagnosed in the general population. The disease morphology presents variably within affected individuals from sudden cardiac death, heart failure, and arrhythmias to those that are asymptomatic [1]. Most of these individuals are asymptomatic throughout their lifetime, which often leads to a delay in diagnosis. Roughly 20 million people throughout the world are affected by hypertrophic cardiomyopathy (HCM), only 10% of cases are identified, and only 6% are symptomatic [1,2]. A vast majority of the literature primarily focuses on cases diagnosed in pediatric, adolescent, and young adult populations. The elderly population is more likely to be an undiagnosed subset of patients with HOCM [3]. The purpose of this case presentation is to illustrate a case of HOCM diagnosed through point-of-care ultrasound (POCUS) in the emergency department (ED) in an elderly male presenting with undifferentiated syncope.

## Case presentation

An 86-year-old male with a past medical history of hypertension, diabetes, and coronary artery disease presented to the ED after a syncopal event at home that was witnessed by his family. The patient did not recall any trauma. The patient’s daughter stated that he had complained of chest pain on a phone call with her the night before. He denied fever, nausea, vomiting, shortness of breath, abdominal pain, or diaphoresis. He did not have any recent surgeries or periods of prolonged immobility. There was no family history of sudden cardiac death. The patient was asymptomatic upon arrival to the ED.

**Figure 1:**
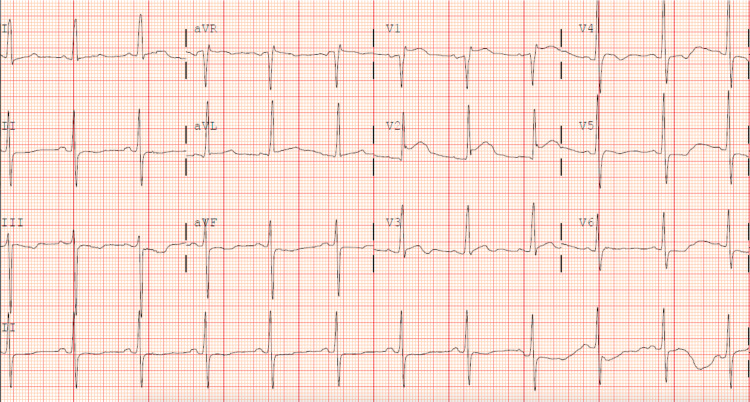
Sinus rhythm with left ventricular hypertrophy

The patient’s initial vital signs were as follows: temperature of 98.2°F, heart rate of 70, respiratory rate of 18, blood pressure of 132/65, and an oxygen saturation of 98% on room air. He was well appearing, non-toxic, and in no acute distress. A cardiac exam upon auscultation showed a regular rate and rhythm and a mid-systolic murmur over the left sternal border. The rest of his physical exam was unremarkable. The patient’s electrocardiogram (ECG) (Figure 1) showed sinus rhythm with left ventricular hypertrophy (LVH). In this ECG, the Modified Cornell Criteria for LVH is fulfilled in that the R-wave in aVL being greater than 12 mm in amplitude; however, it does not fulfill the Sokolow-Lyon Criteria for LVH in that the sum of the S-wave in V1 plus the R wave in V5 or V6 is not greater than 35 mm.

The patient’s abnormal lab results included two cardiac troponins that both measured 0.05 (with the normal range of our institution being <0.04), hemoglobin of 11.1, and the basic metabolic panel was within normal limits except for slight elevation in blood urea nitrogen (BUN) to 41 and creatinine of 1.2. POCUS was performed shortly after the ECG and physical exam, and the following video clips were obtained (Videos 1, 2).

**Video 1:** Apical four-chamber view demonstrating severe asymmetric septal wall thickening with significantly decreased left ventricular cavity size

**Video 2:** Parasternal short-axis view demonstrating a hypertrophied left ventricle with decreased left ventricular cavity size

The chest x-ray was unremarkable with no evidence of pulmonary edema or pleural effusion. Computed tomography of the head showed no mass, hemorrhage, or acute infarct.

Based on the bedside ultrasound findings, the cardiology team was consulted, and the patient was admitted to the cardiac telemetry unit with the diagnosis of syncope, HOCM, and NSTEMI. The patient was emergently assessed by electrophysiology and cardiology teams, for placement of ICD, loop recorder, and consideration of surgical myomectomy or alcohol ablation therapy. The patient had a comprehensive echocardiogram performed by the cardiology team, which confirmed the POCUS findings: small left ventricle size and severe asymmetric LVH with mild resting left ventricular outflow tract obstruction (LVOTO) suggestive of HOCM. Ultimately, the patient was discharged from the hospital with a loop recorder and close cardiology follow-up.

## Discussion

HOCM has an annual mortality rate of about 0.5% and predisposes patients to arrhythmias, particularly atrial fibrillation, sudden cardiac death, cardiac ischemia, and systolic heart failure [4]. Non-obstructive cardiomyopathy has been shown to have a non-negligible influence on long-term mortality, which is not significantly different when compared to HOCM long-term mortality. Non-obstructive HCM predisposes the patient to ventricular tachycardia, ventricular fibrillation, and microvascular ischemia [5]. Diagnosis of this disease is critical in both non-obstructive and obstructive variants. HCM ultrasound criteria in adults include: septal wall thickening ≥15 mm or septal measurement in conjunction with a non-dilated left ventricular chamber or a septal posterior wall-thickness ratio of >1.3 in normotensive patients, or septal to posterior wall-thickness ratio >1.5 in hypertensive patients [4,6]. Cardiac anatomic abnormalities are frequently lower on the differential diagnosis for syncope in the elderly population. Undifferentiated syncope in an elderly patient has a vast differential diagnosis involving many different organ systems and pathologies including, but not limited to, neurologic, cardiac, metabolic, pulmonary, toxicological, and infectious etiologies.

As much of the diagnostic imaging and labs were unremarkable in the case, the patient most likely had a syncopal episode due to left ventricular outflow obstruction or arrhythmia in the setting of HOCM. Left ventricular outflow obstruction arises from a combination of both a fixed LVH of the septum and dynamic systolic anterior motion of the mitral valve leaflets [6]. Left ventricular outflow tract obstruction can lead to left ventricular remodeling due to increased ventricular wall stress, predisposing them to ischemia and ventricular arrhythmias [7]. These individuals have a higher likelihood of suffering from other comorbidities such as congestive heart failure, arrhythmia, and hypovolemia that could predispose them to worsen outflow obstruction, resulting in syncope. Any traumatic injury that is sustained from a fall due to a syncopal episode could have a devastating impact on the rest of their life. It is important to make this diagnosis in a timely manner especially in the elderly population.

## Conclusions

Ultrasound is a rapid and readily available tool that can be used for the immediate assessment of a patient with undifferentiated syncope. The use of ultrasound, in this case, expedited diagnosing the correct pathology and led the patient to receive the appropriate medical interventions to reduce the risk of sudden death and significant morbidity. This case indicates that even within the elderly population, anatomic cardiac abnormalities should be considered, and POCUS can provide crucial information leading to an accurate diagnosis.
